# Supplementary figures and images for: Correlation of a commercial platform’s results with post-vaccination SARS-CoV-2 neutralizing antibody response and clinical host factors
Source: PLoS One. 2023 Aug 29;18(8):e0289713. doi: 10.1371/journal.pone.0289713 (PMC10464955; doi:10.1371/journal.pone.0289713)

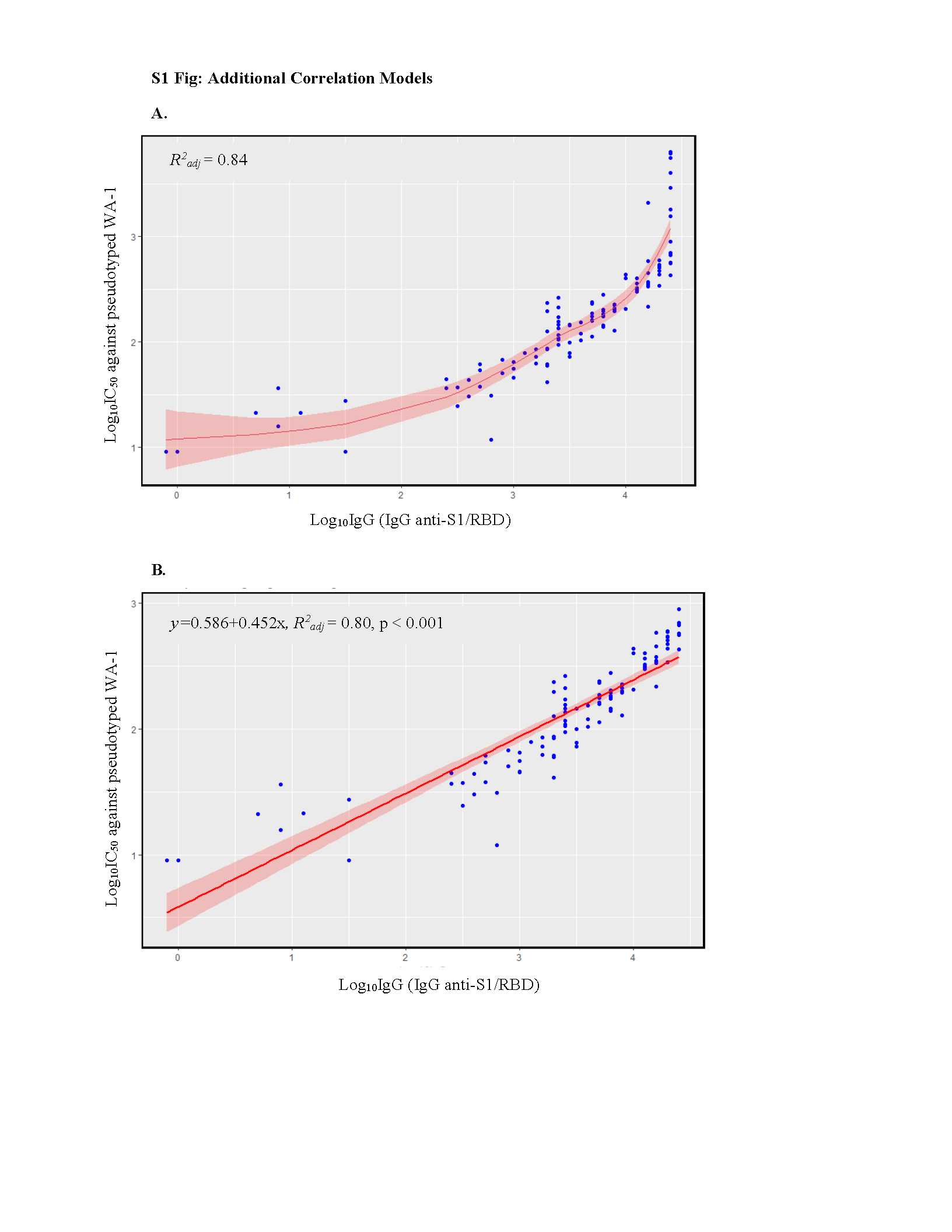

Supplement: S1 Fig — (A) Loess Correlation Between Log10IC50 and Log10IgG. The Loess (locally estimated scatterplot smoothing) is a non-linear correlation model which yields an adjusted R2 of 0.84. We illustrate this model as a supplement because it does not produce a mathematical formula which would allow for easy replication and use, but does demonstrate the possibility of establishing an even stronger correlation for future applications. (B) Linear Correlation Between Log10IC50 and Log10IgG without IgG values over the detection limit. A sensitivity analysis examined the correlation if any values that were above the limit of detection (read as >25,000) on the commercial platform we used were removed. This sensitivity analysis demonstrated an improvement in the adjusted R-squared value of the correlation to 0.80. (TIF) [file pone.0289713.s001.tif]

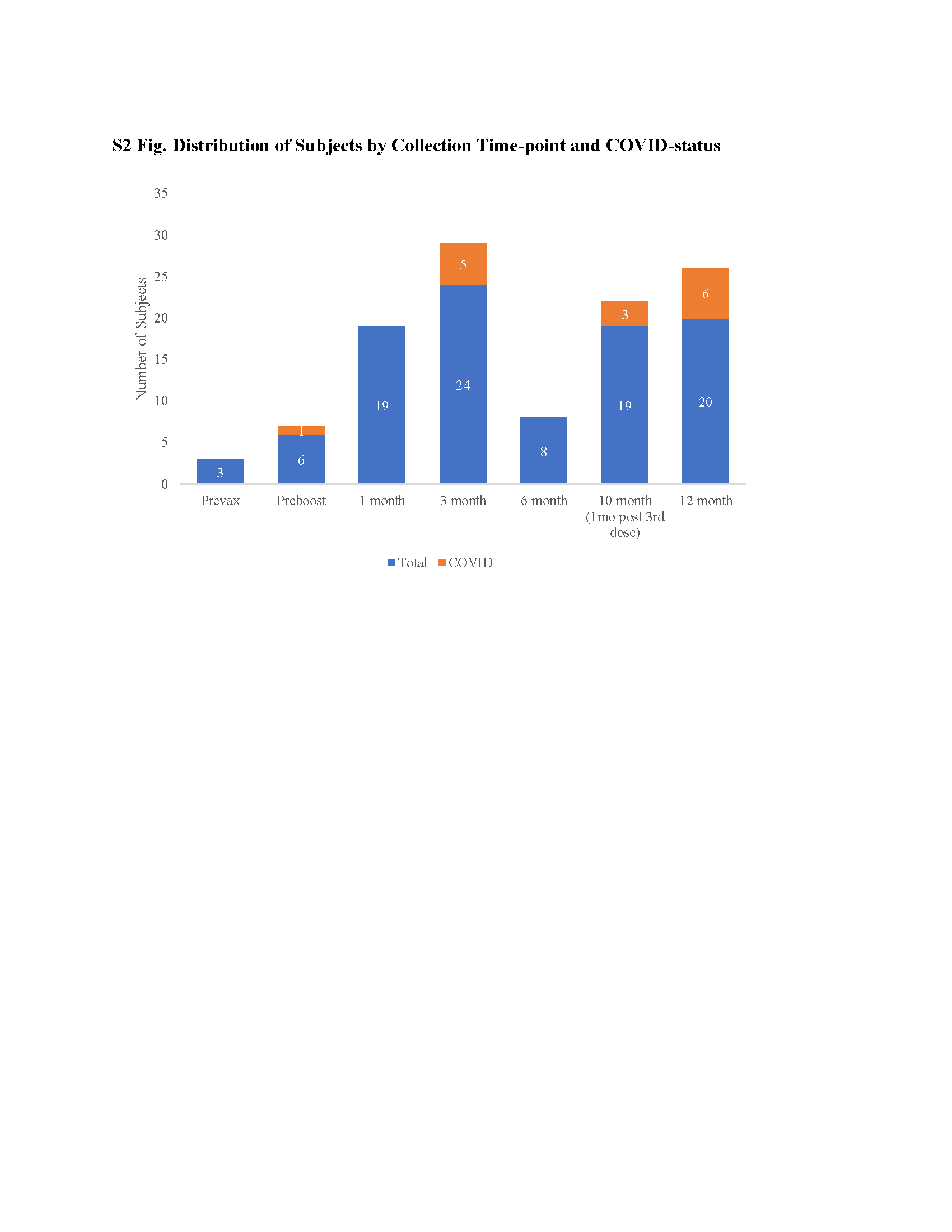

Supplement: S2 Fig — COVID-positivity was defined as RT-PCR COVID- diagnosis at any time prior to collection. (TIF) [file pone.0289713.s002.tif]
