# Supplementary material for: Correlation of a commercial platform’s results with post-vaccination SARS-CoV-2 neutralizing antibody response and clinical host factors
Source: PLoS One. 2023 Aug 29;18(8):e0289713. doi: 10.1371/journal.pone.0289713 (PMC10464955; doi:10.1371/journal.pone.0289713)
Supplement: Attachment — Submitted filename: PONE-D-23-08631 review.docx [file pone.0289713.s004.docx]

**Reviewer's recommendations**

1. As it is known, antibody titers spontaneously decrease over time, and also may increase due to COVID-19 or vaccination. For this reason, to evaluate the correlations of the neutralizing antibody IC50 measurement and the anti-RBD-S1-IgG (IgG) antibody measurement, they must be studied from the sera samples taken at the same time-point. In order to indicate that the study was designed in this way and to make the study workflow easier to understand, it would be beneficial to use a visual abstract showing at which time-points, how many people in which groups (prior COVID infection/or not, etc.), and which tests were studied.
2. Could you explain the term 'intercept' and its purpose of usage in Table-1? What do the obtained values mean and how are they interpreted?
